# Bone and Mineral Metabolism in Patients with Primary Aldosteronism

**DOI:** 10.1155/2014/836529

**Published:** 2014-04-03

**Authors:** Luigi Petramala, Laura Zinnamosca, Amina Settevendemmie, Cristiano Marinelli, Matteo Nardi, Antonio Concistrè, Francesco Corpaci, Gianfranco Tonnarini, Giorgio De Toma, Claudio Letizia

**Affiliations:** ^1^Internal Medicine and Secondary Hypertension Unit, Department of Internal Medicine and Medical Specialties, University of Rome La Sapienza, Viale del Policlinico 155, 00165 Rome, Italy; ^2^Department of Surgery, P.Valdoni, University of Rome La Sapienza, Viale del Policlinico 155, 00165 Rome, Italy

## Abstract

Primary aldosteronism represents major cause of secondary hypertension, strongly associated with high cardiovascular morbidity and mortality. Aldosterone excess may influence mineral homeostasis, through higher urinary calcium excretion inducing secondary increase of parathyroid hormone. Recently, in a cohort of PA patients a significant increase of primary hyperparathyroidism was found, suggesting a bidirectional functional link between the adrenal and parathyroid glands. The aim of this study was to evaluate the impact of aldosterone excess on mineral metabolism and bone mass density. In 73 PA patients we evaluated anthropometric and biochemical parameters, renin-angiotensin-aldosterone system, calcium-phosphorus metabolism, and bone mineral density; control groups were 73 essential hypertension (EH) subjects and 40 healthy subjects. Compared to HS and EH, PA subjects had significantly lower serum calcium levels and higher urinary calcium excretion. Moreover, PA patients showed higher plasma PTH, lower serum 25(OH)-vitamin D levels, higher prevalence of vitamin D deficiency (65% versus 25% and 25%; *P* < 0.001), and higher prevalence of osteopenia/osteoporosis (38.5 and 10.5%) than EH (28% and 4%) and NS (25% and 5%), respectively. This study supports the hypothesis that bone loss and fracture risk in PA patients are potentially the result of aldosterone mediated hypercalciuria and the consecutive secondary hyperparathyroidism.

## 1. Introduction

Primary aldosteronism (PA) is a condition caused by overproduction of aldosterone and is a major cause of secondary hypertension accounting for 0.5–13% of all hypertensive subjects [1]. In a large prospective study of 1.180 Italian patients with newly diagnosed arterial hypertension (known by the acronym PAPY), primary aldosteronism was diagnosed in 11% of patients [2]. The two main causes of PA are aldosterone-producing adenoma (APA) and bilateral adrenal hyperplasia, so called idiopathic hyperaldosteronism (IHA) [3].

Patients with PA typically present with hypertension, high plasma aldosterone concentrations (PAC) that are typically associated with a low plasma rennin activity levels (PRA), and varying degrees of hypokaliemia and metabolic alkalosis. PA is strongly associated with an excess of cardiovascular morbidity and mortality risk that cannot be explained only by arterial hypertension [4].

In recent decades, dynamic studies have demonstrated multiple biological properties of aldosterone, exceeding its classic effect on the water and electrolyte balance. In fact, excess of aldosterone secretion exerts proinflammatory effects, vascular and renal fibrosis, and actions of some cytokines and influences the immune system [5, 6].

Besides cardiovascular and metabolic alterations experimental studies in rats showed that aldosterone excess may also impact mineral homeostasis. In particular, hyperaldosteronism is reported to elevate urinary calcium excretion [7], and urinary calcium correlates with sodium excretion; each 100 mEq/dL increment sodium excretion promotes an increase of 40 mg/dL in calcium excretion [8]. Increased urinary calcium excretion in hyperaldosteronism could be due to the reduced reabsorption on sodium in aldosterone-insensitive tubular sites [9]. Moreover, prolonged ipercalciuria in PA can determine secondary increase of parathyroid hormone (PTH), by the chief cells of the parathyroid gland, a principal regulatory of calcium and phosphate homeostasis.

Recently, in a relative cohort of patients with unequivocally confirmed PA due to APA, Maniero et al. [10] showed a highly significant 31% increase in the number of cases of hyperparathyroidism, thus suggesting that there is a bidirectional functional link between the adrenocortical zona glomerulosa and the parathyroid gland. Moreover, these researchers demonstrated the expression of the mineral corticoid receptors (MR) in both PTH secreting adenoma and in parathyroid tissue [11], and the MR was predominantly located in the nucleus of the parathyroid cells, indicating that aldosterone participate in a “tonic” regulation of PTH synthesis and secretion. Finally, Tomaschitz et al. [7] showed that patients with PA are with secondary hyperparathyroidism that can be successfully treated with either mineral corticoid receptor antagonists or adrenal surgery.

The aim of this study was to evaluate the impact of aldosterone excess on mineral metabolism and bone mass density (BMD) in patients with PA.

## 2. Materials and Methods

### 2.1. Subjects

We enrolled 73 consecutive PA patients referred to the Internal Medicine and Secondary Hypertension Unit, Department of Internal Medicine and Medical Specialties, University of Rome La Sapienza, Italy, from 2009 to 2012.

Patients were divided according to the subtypes into 2 subgroups: APA (35 pts) and IHA (38 pts), matched for age, sex, and blood pressure values.

Patients with renal failure were not included in this study, and all patients were on a normal sodium/potassium restrictions. Control group consists of patients with essential hypertension (EH) and healthy subjects (HS). Previous antihypertensive therapy was withdrawn in all hypertensive patients at least two weeks (in case of spironolactone at least 4 weeks) before the investigation. To standardize the treatment and to eliminate the interference of antihypertensive drugs, all patients were switched to alpha-blocker (doxazosin) and slow-releasing calcium channel blocker (verapamil). Patients with hypokaliemia have continued with oral potassium supplementation.

The suspicion of PA was based on the findings of aldosterone-renin ratio (ARR) ≥ 30 (ng/dL)/(ng/mL/h), plasma renin activity (PRA) ≤ 0.2 ng/mL/h, and plasma aldosterone >15 ng/dL, and the diagnosis of PA was confirmed by the lack of aldosterone suppression (<7 ng/dL) following intravenous saline load (2 lt of 0.9% saline infused over 4 hours).

Differential diagnosis of PA forms (APA and IHA) was supported by a computed tomography scan (CT) or magnetic resonance imaging (MRI), and by a selective adrenal venous sampling (AVS). We used AVS criteria according to previously published guidelines [12]; selectivity was defined as adrenal vein/inferior vena cava cortisol gradient >2 and the lateralization was considered to be present when the aldosterone/cortisol ratio at one side was 2 times greater than in contralateral vein [13]. In addition, the diagnosis of APA was confirmed when successful laparoscopic adrenalectomy was done with histological verification of adrenocortical adenoma. Immunohistochemistry was not performed because it is not necessary for the diagnosis.

EH was established after exclusion of secondary hypertension on the basis of appropriate clinical and laboratory evaluation, including PAC/PRA ≤ 30 (ng/dL)/(ng/mL/h) Table 4.

### 2.2. Anthropometric Parameters

All subjects underwent assessment of weight (kg), height (cm), and body mass index (BMI) calculated by the formula (kg/m^2^), waist circumference (WC, cm) measured to a minimum of inspiration to the mid-point of the line joining the last rib and the iliac crest. Office blood pressure (BP) was measured with a standard aneroid manometer with subjects sitting for 5 minutes, systolic blood pressure (SBP) was taken as the first sound was on of the cuff (Korotkoff phase I), and diastolic blood pressure (DBP) was taken on the complete disappearance of Korotkoff sounds (phase V). Hypertension was confirmed by repeated BP measurements SBP ≥ 140 mmHg and DBP ≤ 90 mmHg.

### 2.3. Biochemical Parameters

Biochemical variables were determined after an overnight fast by anaerobic sampling and evaluating calcium metabolism, renal function, and lipid-glucose metabolism. Ionized calcium was measured with an analyzer: the range of this method was 1.17–1.33 mmol/l. Intact serum PTH (i-PTH) was measured using a radioimmunoassay method (RIA commercial kits: PTH, Still Water, MN, USA).

Measurement of 25-hydroxyvitamin D [25(OH)D] was performed by means of a chemiluminescence assay (IDS-iSYS 25-hydroxyvitamin D; Immunodiagnostic systems Ltd., Boldon, UK) on an IDS-iSYS multidiscipline automated analyzer. PRA, PAC, and plasma cortisol (PC) levels were measured as previously described [14].

### 2.4. Bone Mineral Density

Bone mineral density (BMD) at lumbar spine (L1–L4) and femoral neck (FN) was obtained in all subjects using dual-energy X-ray absorptiometry (DEXA) using Hologic QDR-4800 device (Hologic Inc., Walstrom, MA, USA) according to WHO recommendations. The assessment of BMD was expressed as g/m^2^ and as standard deviation from the mean peak bone mass revealed in healthy subjects adults of the same sex (*T*-score).

The diagnosis of osteoporosis was made in the case of *T*-score ≤ 2.5, osteopenia if *T*-score was between 2.5 and 1, and normal bone mass was made with superior *T*-score of 1. Regarding the precision of BMD evaluation, the coefficient variation was 1% at the lumbar spine and 1.2% at the femoral neck site.

This study was conducted in accordance with the Declaration of Helsinki guidelines and also approved by a local ethical committee. All subjects gave their informed consent before the study begun.

### 2.5. Statistical Analysis

Analysis was performed using Sigmastat Program (Jandel Corporation, USA). All data are expressed as mean ± standard deviation (±SD). Comparison for variables between the groups of subjects was performed using Student's *t*-test and Mann-Whitney test for nonparametric variables. Correlations between variables were assessed by simple linear regression analysis. Linear regression analysis was performed to evaluate the relationship among variables in all subjects. *P* value < 0.05 was considered statistically significant.

## 3. Results

The main characteristics of study subjects are summarized in Tables 1, 2, and 3. Seventy-three subjects were affected by PA (51 males, 22 females; mean age 52.5 ± 11.2 yrs); among these, 35 PA patients (48%) had an APA, whereas 28 (52%) patients had an IHA. Seventy-three subjects were affected by EH (35 males, 38 females; mean age 55.6 ± 12.4 yrs), and 40 subjects were normotensive and otherwise healthy (HS) (16 males, 24 females; mean age 55.7 ± 6.1 yrs).

Clinical and biohumoral parameters for each study group are reported in detail in Tables 1 and 2. In particular, PA and EH subjects showed highest BMI and WC (*P* < 0.002; *P* < 0.003, resp.) compared to HS. For these anthropometric parameters, no difference was found in PA patients subgroup (APA and IHA).

As expected, serum potassium, PAC, PRA values, and ARR were significantly different in PA patients when compared with EH and HS subjects (*P* < 0.001 for all).

### 3.1. Mineral Metabolism

All subjects with PA had significant lower serum calcium levels (*P* < 0.001) associated to higher calcium excretion values (*P* < 0.001), compared to EH and HS subjects (Table 2). Moreover, PA patients showed lower serum 25(OH)-vitamin D levels and higher plasma PTH values with respect to EH and HS subjects (*P* < 0.001 and *P* < 0.001, resp.) (Table 2). No statistically significant difference for these mineral parameters was showed in PA subtypes patients (APA and IHA).

**Figure 1:**
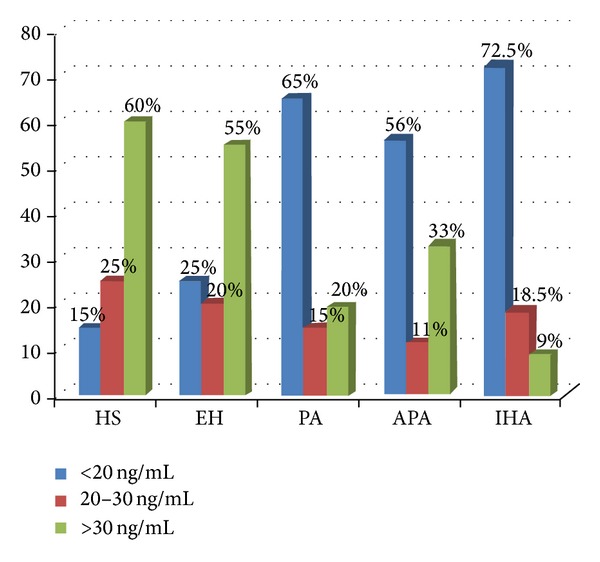
Plasma levels of 25(OH)-vitamin D in all subjects enrolled. HS: healthy subjects; EH: essential arterial hypertension; PA: primary aldosteronism; APA: aldosterone-producing adrenal adenoma; IHA: idiopathic bilateral hyperplasia.

Several studies have shown that vitamin D scoreis easily assessed by serum 25(OH)-vitamin D. Concentration less than 20 ng/mL is generally considered vitamin D deficiency whereas between 20 and 30 ng/mL vitamin insufficiency. In Figure 1, we reported the behavior of serum 25(OH)-vitamin. D levels in all study groups. In particular the prevalence of vitamin D deficiency was significantly higher in patients with PA than in EH and HS subjects (65% versus 25% and 25%, resp.; *P* < 0.001). Among PA subjects the prevalence of vitamin D deficiency was higher in subjects with IHA when compared with those with APA (72.5% versus 56%; *P* < 0.01, resp.).

### 3.2. Bone Densitometry (BMD)

**Figure 2:**
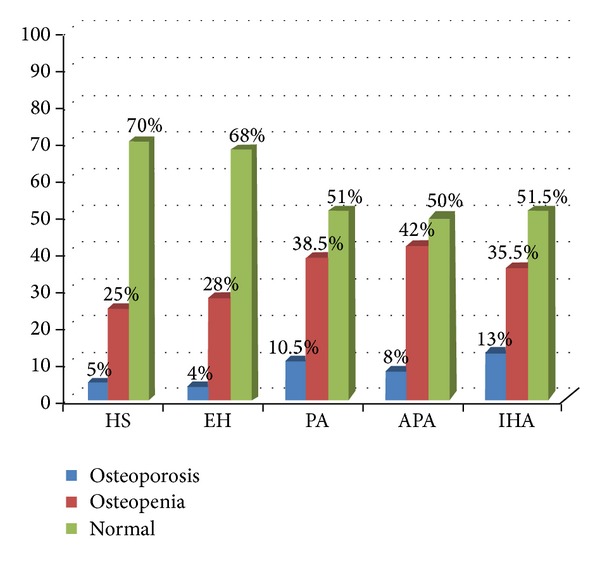
Prevalence of osteoporosis in in all subjects enrolled. HS: healthy subjects; EH: essential arterial hypertension; PA: primary aldosteronism; APA: aldosterone-producing adrenal adenoma; IHA: idiopathic bilateral hyperplasia.

Bone densitometric parameters are reported in Table 4. In PA patients we found high prevalence of osteopenia and osteoporosis (38.5 and 10.5%) than EH (28% and 4%) and NS (25% and 5%), respectively. Moreover, PA patients with APA showed more bone remodeling with respect to IHA patients (Figure 2). Study of correlations revealed in all patients with PA a negative correlation with PAC and BMD Neck (*r* = −0.27; *P* < 0.05) and with the *T*-score Neck (*r* = −0.28; *P* < 0.04) (Table 5).

## 4. Discussion

PA is the most common form of hormone related arterial hypertension, representing a curable disease [1]. PA is characterized by autonomous overproduction of adrenal aldosterone with suppression of PRA, sodium retention, and consequent hypertension. Various primary adrenal processes cause this syndrome. Some of them are best treated by surgery and others by medicine [3]. In the past it had been documented that PA contributed to the development of cardiovascular disease [15], and the metabolic alterations caused by inappropriate secretion of aldosterone are being recognized such as metabolic syndrome [16]. However, only recently the calcium metabolism alterations and hyperaldosteronism have been systematically recognized [11]. In particular, studies aimed to verify the hypothesis postulating the effect of aldosterone on the secretion of PTH are also worth mentioning [10]. In this study we examined the calcium mineral metabolism and BMD in PA patients, (both APA and IHA), compared to EH patients. Our results showed that all PA patients had significant lower serum calcium levels associated to higher calcium excretion compared to EH. Moreover, PA patients showed lower serum 25(OH)-vitamin D levels and higher plasma PTH values with respect to EH. No statistically significant differences for these mineral parameters were showed in PA subtypes patients (APA versus IHA). These data confirmed and extend other results reported in the literature [17–19].

In particular, in a study firstly conducted in humans, patients with PA were detected to have significantly higher concentrations of PTH compared to both normal and hypertensive subjects [20]. In Graz Endocrine Causes of Hypertension (GECOH) study, Pilz and coworkers [17] reported in 10 PA patients (5 APA and 5 IHA) lower serum calcium and higher plasma PTH levels compared to EH patients; however, serum 25(OH)-vitamin D concentrations were similar in both groups. These authors hypothesized that PA contributes to secondary hyperparathyroidism.

Recently, Ceccoli and coworkers [18] in 116 PA patients (40 with APA and 70 with IHA) compared with 110 EH patients showed an increase of PTH levels and urinary calcium excretion, while serum calcium decreases with comparable vitamin D levels. Moreover, this PTH increase, more evident in patients with APA than in those with IHA, is reversible after appropriate treatment of aldosterone excess. These data supported the hypothesis that secondary hyperparathyroidism in PA seems to be due to the presence of aldosterone excess with an increased urinary excretion of calcium and consequent hypocalcaemia. In fact, renal hypercalciuria in these patients may be due to aldosterone excess combined with high salt retention. Expansion of effective circulating value in PA patients decreases proximal tubule reabsorption of calcium as well as sodium because calcium reabsorption is coupled to transtubular sodium uptake [18].

Although the distal nephron can reabsorb calcium, excessive delivery can overwhelm this absorptive capacity. In addition, potassium depletion causes intracellular acidosis [9]; aldosterone, by augmenting sodium chloride cotransfer in the distal nephron [21], can lead indirectly to impaired calcium reabsorption. The reduction in urine calcium excretion in response to specific treatment (adrenalectomy or MR-antagonists) reported by Ceccoli et al. [18] in patients with PA supported the hypothesis that hyperaldosteronism is the main cause of the hypercalciuria in these patients. Another data shown in our study is the alterations of BMD evaluated as *T*-score value for two skeletal sites (the lumbar spine and femoral neck) and higher prevalence of osteoporosis and osteopenia in PA patients with respect to EH and HS. Our data are in line with previous studies [17, 22].

In the setting of PA, bone metabolism might be affected (1) directly by aldosterone-MR-mediated effects on osteoblasts and osteoclasts and (2) indirectly by PTH levels and increased bone resorption [20]. Salcuni et al. [19] showed higher 24 h urinary calcium and elevated PTH levels in patients with PA compared to patients with adrenal incidentaloma without aldosterone excess. Moreover, these authors documented significantly lower bone mineral density (measured at the lumbar spine, total, and femoral neck) in PA patients compared to patients without hyperaldosteronism. Moreover, Ceccoli et al. [18] reported in 40 patients with PA (16 APA and 24 IHA), available both at baseline and after adrenalectomy (APA) and treatment with MR antagonists (IHA), an improvement of BMD and suggested that these changes seem to be due to target treatment and not the vitamin D status, because there were no significant changes in serum 25(OH)-vitamin D before and after treatment of aldosterone excess.

In our study, whereas, we found lower serum 25(OH)-vitamin D levels associated at the higher available phosphate levels in PA patients compared with EH patients and a higher percentage of vitamin D deficiency (<20 ng/dL). This is an important finding, because the hypovitaminosis D can favor bone abnormalities and reduced bone mass and associated a significant increase in renal excretion and increased circulating levels of PTH, leading to typical feature of osteomalacia.

In conclusion, these observations support the hypothesis that bone loss and potentially fracture risk in PA patients are potentially the result of aldosterone mediated hypercalciuria and the consecutive secondary hyperparathyroidism.

## Figures and Tables

**Table 1:** Demographic and anthropometric parameters in all subjects enrolled.

Patient	Years (yrs)	BMI (Kg/m^2^)	Waist circumference (cm)	SBP (mmHg)	DBP (mmHg)
PA (n.73)	52.5 ± 11.2	28.2 ± 4.7*	99.8 ± 13.1*	138.3 ± 16.8*	85.9 ± 11.4*
EH (n.73)	55.6 ± 12.4	29 ± 5*	100.5 ± 11.2*	131 ± 18.8*	82.4 ± 11.2*
HS (n.40)	55.7 ± 6.1	25.1 ± 2.2	95.5 ± 6.8	119.1 ± 4.2	77.2 ± 5.1
*P*	ns	<0.002 versus HS	<0.003 versus HS	<0.01 versus HS	<0.01 versus HS

APA (n.35)	52.8 ± 11.5	27.6 ± 4.8	100.4 ± 12.9	138.8 ± 19.1	88.3 ± 9.6
IHA (n.38)	52.5 ± 11.2	28.6 ± 4.6	99.3 ± 13.6	137.3 ± 14.5	83.4 ± 9.6
*P*	ns	ns	ns	ns	ns

PA: primary aldosteronism; EH: essential arterial hypertension, HS: healthy subjects; APA: aldosterone-producing adrenal adenoma; IHA: idiopathic bilateral hyperplasia; BMI: body mass index; SBP: systolic blood pressure; DBP: diastolic blood pressure.

*P value.

**Table 2:** Biochemical parameters of all subjects enrolled.

Patient	Serum creatinine (mg/dL)	K (mEq/L)	Ca (mg/dL)	Ca^2+^ (mmol/L)	Ca-Ur (mg/24 h)	P (mg/dL)	PTH (pg/mL)	ALP (UI/L)	25-OH vitamin D (ng/mL)
PA (n.73)	0.9 ± 0.2	3.8 ± 0.5*	9.2 ± 0.4*	1.2 ± 0.09	242.8 ± 116.7*	3.5 ± 0.6	48.9 ± 19.9*	163.3 ± 33.9*	17.8 ± 12.5*
EH (n.73)	1.02 ± 0.2	4.2 ± 0.4	9.7 ± 0.3	1.2 ± 0.03	164.1 ± 84*	3.4 ± 0.4	30.7 ± 11.9	87.4 ± 46.7	32.9 ± 16
HS (n.40)	0.88 ± 0.2	4.17 ± 0.4	9.4 ± 0.3	1.21 ± 0.02	154.6 ± 17.3	3.4 ± 0.3	29.1 ± 2.4	100.3 ± 52.8	23.8 ± 12.8
*P*	ns	<0.001 versus EH-HS	<0.001 versus EH-HS	ns	<0.001 versus EH-HS	ns	<0.001 versus EH-HS	<0.001 versus EH-HS	<0.001 versus EH-HS

APA (n.35)	0.9 ± 0.2	3.7 ± 0.7	9.2 ± 0.5	1.2 ± 0.07	222.5 ± 100.7	3.4 ± 0.7	46 ± 20.1	179.1 ± 27.4	21.3 ± 16.6
IHA (n.38)	0.8 ± 0.2	3.9 ± 0.3	9.2 ± 0.4	1.2 ± 0.12	274.7 ± 140.4	3.6 ± 0.6	50.6 ± 20.2	135.7 ± 26.9	18.5 ± 17.8
*P*	ns	ns	ns	ns	ns	ns	ns	ns	ns

K: serum potassium; Ca: serum total calcium; Ca^2+^: ionized serum calcium; Ca-Ur: 24-hour urinary calcium excretion; P: serum phosphorus; PTH: parathyroid hormone; ALP: alkaline phosphatase.

*P value.

**Table 3:** Renin-angiotensin-aldosterone system parameters in all subjects enrolled.

Patient	PAC (ng/dL)	PRA (ng/mL/h)	PA/PRA ratio (ng/mL : ng/mL/h)	PAC postinfusion test (ng/dL)	AUR (*μ*g/24 h)
PA (n.73)	37 ± 25.1*	0.9 ± 0.7*	41.1 ± 11.5*	115.9 ± 78.7*	31.6 ± 18.1*
EH (n.73)	22.5 ± 13	1.4 ± 1.6	16.7 ± 7.3	24.5 ± 8.7	16.3 ± 4.5
HS (n.40)	9.2 ± 1.7	1.1 ± 0.4	8.4 ± 2.8	—	18.3 ± 5.3
*P*	<0.001 versus EH-HS	<0.001 versus EH	<0.001 versus EH-HS	<0.001 versus EH	<0.001 versus EH-HS

APA (n.35)	39.8 ± 25.6	0.7 ± 0.6	56.9 ± 15.2	148.1 ± 95.7	34.3 ± 22.8
IHA (n.38)	34.4 ± 24.6	1.1 ± 0.8	31.3 ± 5.6	85.1 ± 40.5	29.4 ± 12.8
*P*	ns	ns	ns	<0.001	ns

PA: primary aldosteronism; EH: essential arterial hypertension, HS: healthy subjects; APA: aldosterone-producing adrenal adenoma; IHA: idiopathic bilateral hyperplasia; PAC: plasma aldosterone concentration; PRA: plasma renin activity; AUR: 24-hour aldosterone urinary excretion.

*P value.

**Table 4:** Bone mineral density (BMD) evaluated by dual-energy X-ray absorptiometry (DXA) in all subjects enrolled.

Patient	*T*-score L1–L4	BMD L1–L4 (g/cm^2^)	*T*-score FN	BMD FN (g/cm^2^)
PA (n.73)	−0.28 ± 1.3*	1.01 ± 0.17*	−0.67 ± 1.1*	0.84 ± 0.16
EH (n.73)	0.03 ± 0.6	1.11 ± 0.17	−0.29 ± 0.7	0.84 ± 0.12
HS (n.40)	0.027 ± 0.8	1 ± 0.09	−0.30 ± 0.6	0.81 ± 0.08
*P*	0.06* versus EH-HS	0.06* versus EH-HS	0.06* versus EH-HS	ns

APA (n.35)	−0.30 ± 1.3	1 ± 0.18	−0.7 ± 1.05	0.82 ± 0.14
IHA (n.38)	−0.25 ± 1.4	1.02 ± 0.17	−0.63 ± 1.3	0.85 ± 0.19
*P*	ns	ns	ns	ns

PA: primary aldosteronism; EH: essential arterial hypertension, HS: healthy subjects; APA: aldosterone-producing adrenal adenoma; IHA: idiopathic bilateral hyperplasia; L1–L4: lumbar spine side; FN: femoral neck side.

**Table 5:** Study correlation in PA subjects.

Parameters	*P*	*r*
24 h calcium excretion		
Serum calcium	<0.01	−0.56
Age	<0.001	−0.75
PTH		
BMD FN	<0.02	−0.461
*T*-score FN	<0.01	−0.2
Serum phosphorus		
BMD L1–L4	<0.03	−0.403
24 h aldosterone urinary excretion	<0.03	−0.37
Plasma aldosterone		
BMD FN	<0.05	−0.27
*T*-score FN	<0.04	−0.28

BMD: bone mineral density; FN: femoral neck side; L1–L4: lumbar spine side.
